# (3-Methyl-3a,4,7,7a-tetra­hydro-5*H*-4,7-methano­isoxazolo[4,5-*d*][1,2]oxazin-5-yl)(phen­yl)methanone

**DOI:** 10.1107/S1600536814007740

**Published:** 2014-04-12

**Authors:** Alan J. Lough, Jaipal R. Nagireddy, William Tam

**Affiliations:** a Department of Chemistry, University of Toronto, Toronto, Ontario, M5S 3H6, Canada; b Department of Chemistry, University of Guelph, Guelph, Ontario, N1G 2W1, Canada

## Abstract

The title compound, C_14_H_14_N_2_O_3_, is the *exo* isomer with a *syn* arrangement of two O atoms in the isoxazole and oxazine rings. The dihedral angle between the isoxazole and phenyl rings is 60.38 (4)°. In the crystal, weak C—H⋯O hydrogen bonds link the mol­ecules, forming a three-dimensional network. The isoxazole O atom is an acceptor for three of these hydrogen bonds.

## Related literature   

For 1,3-dipolar cyclo­addition reactions of symmetrical and unsymmetrical bicyclic alkenes, see: Yip *et al.* (2001 ▶); Mayo *et al.* (2001 ▶). For a related structure, see: Lough *et al.* (2014 ▶).
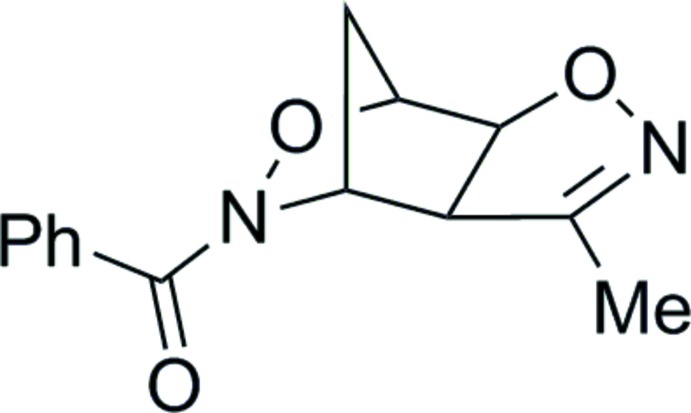


## Experimental   

### 

#### Crystal data  

C_14_H_14_N_2_O_3_
*M* *_r_* = 258.27
Orthorhombic, 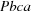

*a* = 9.5030 (18) Å
*b* = 10.2912 (16) Å
*c* = 25.347 (5) Å
*V* = 2478.9 (8) Å^3^
*Z* = 8
Mo *K*α radiation
μ = 0.10 mm^−1^
*T* = 147 K
0.38 × 0.16 × 0.10 mm

#### Data collection  

Bruker Kappa APEX DUO CCD diffractometer
Absorption correction: multi-scan (*SADABS*; Bruker, 2012 ▶) *T*_min_ = 0.671, *T*_max_ = 0.746
12134 measured reflections
2854 independent reflections
2145 reflections with *I* > 2σ(*I*)
*R*_int_ = 0.044

#### Refinement  

*R*[*F* ^2^ > 2σ(*F* ^2^)] = 0.041
*wR*(*F* ^2^) = 0.101
*S* = 1.03
2854 reflections
173 parameters
H-atom parameters constrained
Δρ_max_ = 0.28 e Å^−3^
Δρ_min_ = −0.20 e Å^−3^

### 

Data collection: *APEX2* (Bruker, 2012 ▶); cell refinement: *SAINT* (Bruker, 2012 ▶); data reduction: *SAINT*; program(s) used to solve structure: *SHELXS97* (Sheldrick, 2008 ▶); program(s) used to refine structure: *SHELXL2013* (Sheldrick, 2008 ▶); molecular graphics: *PLATON* (Spek, 2009 ▶); software used to prepare material for publication: *SHELXTL* (Sheldrick, 2008 ▶).

## Supplementary Material

Crystal structure: contains datablock(s) I. DOI: 10.1107/S1600536814007740/is5350sup1.cif

Structure factors: contains datablock(s) I. DOI: 10.1107/S1600536814007740/is5350Isup2.hkl

Supporting information file. DOI: 10.1107/S1600536814007740/is5350Isup3.cml

CCDC reference: 995952

Additional supporting information:  crystallographic information; 3D view; checkCIF report

## Figures and Tables

**Table 1:** Hydrogen-bond geometry (Å, °)

*D*—H⋯*A*	*D*—H	H⋯*A*	*D*⋯*A*	*D*—H⋯*A*
C4—H4*A*⋯O3^i^	1.00	2.50	3.2793 (18)	135
C5—H5*A*⋯O2^ii^	1.00	2.59	3.4017 (18)	138
C7—H7*B*⋯O2^ii^	0.98	2.56	3.3900 (19)	142
C7—H7*C*⋯O2^iii^	0.98	2.60	3.568 (2)	170
C11—H11*A*⋯O3^iv^	0.95	2.58	3.470 (2)	156

Symmetry codes: (i) 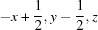; (ii) 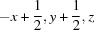; (iii) 
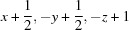
; (iv) 
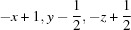
.

**Fig. 1.**
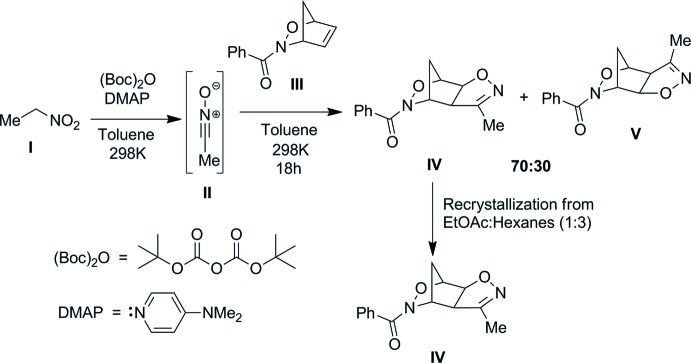
The reaction scheme.

**Fig. 2.**
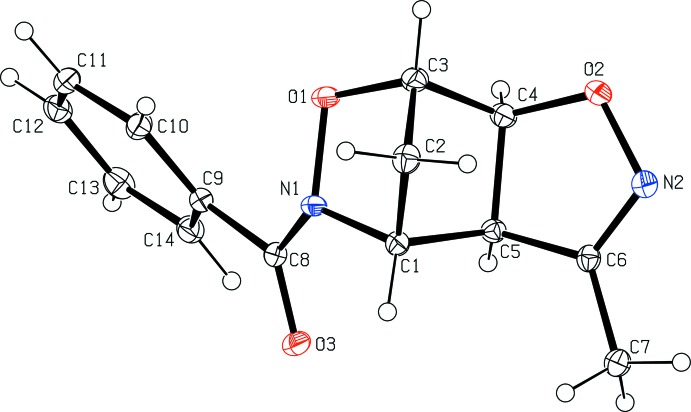
The molecular structure of the title compound showing 30% probability ellipsoids.

**Fig. 3.**
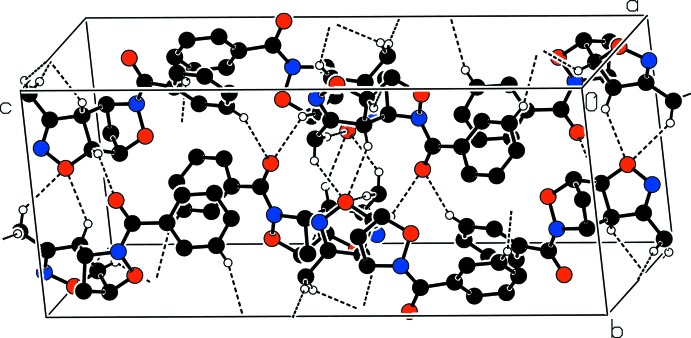
Part of the crystal structure with weak hydrogen bonds shown as dashed lines.

## supplementary crystallographic information

### 1. Comment

We have previously investigated the 1,3-dipolar cycloaddition reactions of symmetrical and unsymmetrical bicyclic alkenes (Yip *et al.*, 2001; Mayo *et al.*, 2001). When expanding this study on *N*-acyl-2-oxa-3-azanorborn-5-enes, the bicyclic alkene (III) reacts (see Fig. 1) with acetonitrile oxide (II) (generated *in situ*) in toluene, to give the cycloadducts (IV) and (V) as regioisomers in the ratio of 70:30 respectively (ratio was determined by isolated yields). The stereochemistry and regiochemistry of the major product (IV) was determined by this single-crystal X-ray analysis. Although different stereoisomers (*exo* and *endo*) could be formed, only the *exo* stereoisomer was formed with a mixture of the corresponding regioisomers. The major product obtained was found to be the *syn* isomer (two O atoms in the rings are on the same side of the molecule).

The molecular structure of the title compound is shown in Fig. 2. The dihedral angle between the isoxazole ring [C4/C5/C6/O2/N2 with r.m.s. deviation 0.0013 Å] and the phenyl ring (C9–C14) is 60.38 (4)°. In the crystal, weak C—H···O hydrogen bonds link molecules forming a three-dimensional network (Fig. 3). The isoxazole O atom is an acceptor for three of these hydrogen bonds. We have prepared by a similar method and carried out the structure determination of a related cycloadduct (Lough *et al.*, 2014)

### 2. Experimental

A solution of nitroethane (I) (126 mg, 0.579 mmol) in toluene (2 ml) was added to a flame-dried flask containing bicyclic alkene (III) (140 mg, 0.642 mmol), (BOC)_2_O (233.7 mg, 1.07 mmol), DMAP (9.4 mg, 0.077 mmol) and toluene (2 ml) *via* a cannula over 10 minutes. The reaction mixture was stirred at room temperature for 18 h. The solvent was removed by rotary evaporation, and the crude product was purified by column chromatography (EtOAc:hexanes = 1:9 to 9:1) to obtain regiosomers (IV) and (V) in 61% and 26% respectively. A solution of isomer (IV) in EtOAc:hexanes = 1:3 gave single crystals suitable for X-ray analysis.

### 3. Refinement

Hydrogen atoms were placed in calculated positions with C—H distances of 0.95–1.00 Å and included in the refinement in a riding-model approximation with *U*_iso_(H) = 1.2*U*_eq_(C) or 1.5*U*_eq_(C_methyl_).

### Crystal data

**Table:**

C_14_H_14_N_2_O_3_	*D*_x_ = 1.384 Mg m^−^^3^
*M**_r_* = 258.27	Mo *K*α radiation, λ = 0.71073 Å
Orthorhombic, *P**b**c**a*	Cell parameters from 2381 reflections
*a* = 9.5030 (18) Å	θ = 2.7–26.8°
*b* = 10.2912 (16) Å	µ = 0.10 mm^−^^1^
*c* = 25.347 (5) Å	*T* = 147 K
*V* = 2478.9 (8) Å^3^	Needle, colourless
*Z* = 8	0.38 × 0.16 × 0.10 mm
*F*(000) = 1088	

### Data collection

**Table:**

Bruker Kappa APEX DUO CCD diffractometer	2145 reflections with *I* > 2σ(*I*)
Radiation source: sealed tube with Bruker Triumph monochromator	*R*_int_ = 0.044
φ and ω scans	θ_max_ = 27.5°, θ_min_ = 1.6°
Absorption correction: multi-scan (*SADABS*; Bruker, 2012)	*h* = −12→9
*T*_min_ = 0.671, *T*_max_ = 0.746	*k* = −11→13
12134 measured reflections	*l* = −24→32
2854 independent reflections	

### Refinement

**Table:**

Refinement on *F*^2^	0 restraints
Least-squares matrix: full	Hydrogen site location: inferred from neighbouring sites
*R*[*F*^2^ > 2σ(*F*^2^)] = 0.041	H-atom parameters constrained
*wR*(*F*^2^) = 0.101	*w* = 1/[σ^2^(*F*_o_^2^) + (0.0436*P*)^2^ + 0.7731*P*] where *P* = (*F*_o_^2^ + 2*F*_c_^2^)/3
*S* = 1.03	(Δ/σ)_max_ < 0.001
2854 reflections	Δρ_max_ = 0.28 e Å^−^^3^
173 parameters	Δρ_min_ = −0.20 e Å^−^^3^

### Special details

**Table:**

Geometry. All e.s.d.'s (except the e.s.d. in the dihedral angle between two l.s. planes) are estimated using the full covariance matrix. The cell e.s.d.'s are taken into account individually in the estimation of e.s.d.'s in distances, angles and torsion angles; correlations between e.s.d.'s in cell parameters are only used when they are defined by crystal symmetry. An approximate (isotropic) treatment of cell e.s.d.'s is used for estimating e.s.d.'s involving l.s. planes.

### Fractional atomic coordinates and isotropic or equivalent isotropic displacement parameters (Å^2^)

**Table:**

	*x*	*y*	*z*	*U*_iso_*/*U*_eq_	
O1	0.42779 (11)	0.16029 (10)	0.34310 (4)	0.0239 (3)	
O2	0.28195 (12)	0.06699 (10)	0.47211 (4)	0.0274 (3)	
O3	0.44907 (11)	0.49825 (10)	0.35864 (4)	0.0261 (3)	
N1	0.48705 (13)	0.28419 (11)	0.35988 (5)	0.0206 (3)	
N2	0.32314 (13)	0.14237 (12)	0.51709 (5)	0.0240 (3)	
C1	0.51026 (15)	0.27468 (14)	0.41673 (6)	0.0196 (3)	
H1A	0.5784	0.3390	0.4316	0.023*	
C2	0.55574 (16)	0.13321 (14)	0.42059 (6)	0.0247 (3)	
H2A	0.6441	0.1146	0.4013	0.030*	
H2B	0.5618	0.1013	0.4574	0.030*	
C3	0.42502 (16)	0.08599 (14)	0.39177 (6)	0.0229 (3)	
H3A	0.4207	−0.0103	0.3867	0.027*	
C4	0.30441 (16)	0.14184 (14)	0.42428 (6)	0.0218 (3)	
H4A	0.2162	0.1520	0.4031	0.026*	
C5	0.36368 (14)	0.27359 (13)	0.44313 (6)	0.0183 (3)	
H5A	0.3038	0.3490	0.4324	0.022*	
C6	0.36722 (14)	0.25337 (14)	0.50174 (6)	0.0193 (3)	
C7	0.41835 (16)	0.35181 (15)	0.54006 (6)	0.0242 (3)	
H7A	0.4135	0.3160	0.5758	0.036*	
H7B	0.3593	0.4297	0.5379	0.036*	
H7C	0.5160	0.3747	0.5318	0.036*	
C8	0.44268 (15)	0.39406 (14)	0.33502 (6)	0.0198 (3)	
C9	0.40242 (15)	0.38382 (14)	0.27853 (6)	0.0201 (3)	
C10	0.46180 (16)	0.29115 (14)	0.24524 (6)	0.0243 (3)	
H10A	0.5260	0.2289	0.2589	0.029*	
C11	0.42718 (18)	0.28984 (15)	0.19209 (6)	0.0271 (4)	
H11A	0.4685	0.2274	0.1693	0.033*	
C12	0.33260 (17)	0.37935 (15)	0.17233 (6)	0.0283 (4)	
H12A	0.3077	0.3772	0.1361	0.034*	
C13	0.27394 (16)	0.47228 (16)	0.20515 (7)	0.0281 (4)	
H13A	0.2094	0.5340	0.1913	0.034*	
C14	0.30932 (15)	0.47535 (14)	0.25820 (6)	0.0241 (3)	
H14A	0.2701	0.5398	0.2806	0.029*	

### Atomic displacement parameters (Å^2^)

**Table:**

	*U*^11^	*U*^22^	*U*^33^	*U*^12^	*U*^13^	*U*^23^
O1	0.0346 (6)	0.0167 (5)	0.0205 (6)	−0.0054 (4)	−0.0012 (5)	−0.0033 (4)
O2	0.0370 (6)	0.0217 (5)	0.0236 (6)	−0.0101 (5)	0.0037 (5)	−0.0003 (4)
O3	0.0372 (6)	0.0194 (6)	0.0218 (6)	−0.0014 (5)	0.0026 (5)	−0.0042 (4)
N1	0.0278 (6)	0.0165 (6)	0.0177 (6)	−0.0063 (5)	−0.0007 (5)	−0.0024 (5)
N2	0.0275 (6)	0.0226 (7)	0.0220 (7)	−0.0017 (5)	0.0025 (5)	0.0002 (5)
C1	0.0212 (7)	0.0208 (7)	0.0167 (7)	−0.0028 (6)	−0.0001 (6)	−0.0003 (6)
C2	0.0244 (7)	0.0249 (8)	0.0248 (8)	0.0046 (6)	0.0015 (6)	0.0010 (6)
C3	0.0315 (8)	0.0157 (7)	0.0213 (8)	0.0002 (6)	0.0004 (6)	0.0008 (6)
C4	0.0234 (7)	0.0195 (8)	0.0224 (8)	−0.0036 (6)	−0.0005 (6)	0.0005 (6)
C5	0.0199 (7)	0.0167 (7)	0.0182 (7)	0.0003 (5)	−0.0011 (6)	0.0002 (6)
C6	0.0188 (6)	0.0202 (7)	0.0187 (8)	0.0025 (5)	0.0015 (6)	0.0013 (5)
C7	0.0292 (8)	0.0254 (8)	0.0179 (8)	−0.0026 (6)	−0.0018 (6)	0.0007 (6)
C8	0.0208 (7)	0.0195 (8)	0.0193 (8)	−0.0013 (6)	0.0044 (6)	−0.0019 (6)
C9	0.0233 (7)	0.0189 (7)	0.0181 (8)	−0.0033 (6)	0.0021 (6)	−0.0003 (6)
C10	0.0315 (8)	0.0219 (8)	0.0194 (8)	0.0016 (6)	0.0027 (6)	−0.0006 (6)
C11	0.0385 (9)	0.0226 (8)	0.0203 (8)	−0.0030 (7)	0.0042 (7)	−0.0029 (6)
C12	0.0353 (8)	0.0288 (9)	0.0206 (8)	−0.0115 (7)	−0.0043 (7)	0.0034 (6)
C13	0.0266 (8)	0.0279 (9)	0.0299 (9)	−0.0018 (6)	−0.0051 (7)	0.0072 (7)
C14	0.0245 (7)	0.0212 (8)	0.0265 (9)	−0.0003 (6)	0.0028 (6)	0.0006 (6)

### Geometric parameters (Å, º)

**Table:**

O1—C3	1.4515 (18)	C5—C6	1.501 (2)
O1—N1	1.4572 (15)	C5—H5A	1.0000
O2—N2	1.4333 (16)	C6—C7	1.485 (2)
O2—C4	1.4524 (18)	C7—H7A	0.9800
O3—C8	1.2296 (17)	C7—H7B	0.9800
N1—C8	1.3613 (19)	C7—H7C	0.9800
N1—C1	1.4611 (18)	C8—C9	1.486 (2)
N2—C6	1.2773 (19)	C9—C14	1.391 (2)
C1—C2	1.522 (2)	C9—C10	1.393 (2)
C1—C5	1.545 (2)	C10—C11	1.387 (2)
C1—H1A	1.0000	C10—H10A	0.9500
C2—C3	1.521 (2)	C11—C12	1.381 (2)
C2—H2A	0.9900	C11—H11A	0.9500
C2—H2B	0.9900	C12—C13	1.385 (2)
C3—C4	1.524 (2)	C12—H12A	0.9500
C3—H3A	1.0000	C13—C14	1.386 (2)
C4—C5	1.544 (2)	C13—H13A	0.9500
C4—H4A	1.0000	C14—H14A	0.9500
			
C3—O1—N1	102.71 (10)	C6—C5—H5A	112.9
N2—O2—C4	109.68 (10)	C4—C5—H5A	112.9
C8—N1—O1	118.17 (11)	C1—C5—H5A	112.9
C8—N1—C1	123.97 (12)	N2—C6—C7	121.22 (14)
O1—N1—C1	106.71 (10)	N2—C6—C5	114.71 (13)
C6—N2—O2	109.36 (12)	C7—C6—C5	124.07 (13)
N1—C1—C2	99.81 (12)	C6—C7—H7A	109.5
N1—C1—C5	106.95 (11)	C6—C7—H7B	109.5
C2—C1—C5	102.78 (11)	H7A—C7—H7B	109.5
N1—C1—H1A	115.2	C6—C7—H7C	109.5
C2—C1—H1A	115.2	H7A—C7—H7C	109.5
C5—C1—H1A	115.2	H7B—C7—H7C	109.5
C3—C2—C1	92.46 (11)	O3—C8—N1	118.93 (13)
C3—C2—H2A	113.2	O3—C8—C9	122.94 (13)
C1—C2—H2A	113.2	N1—C8—C9	117.83 (12)
C3—C2—H2B	113.2	C14—C9—C10	119.79 (14)
C1—C2—H2B	113.2	C14—C9—C8	118.21 (13)
H2A—C2—H2B	110.6	C10—C9—C8	121.87 (13)
O1—C3—C2	103.00 (12)	C11—C10—C9	119.94 (14)
O1—C3—C4	105.92 (11)	C11—C10—H10A	120.0
C2—C3—C4	103.54 (12)	C9—C10—H10A	120.0
O1—C3—H3A	114.4	C12—C11—C10	120.01 (15)
C2—C3—H3A	114.4	C12—C11—H11A	120.0
C4—C3—H3A	114.4	C10—C11—H11A	120.0
O2—C4—C3	111.21 (12)	C11—C12—C13	120.32 (15)
O2—C4—C5	105.13 (12)	C11—C12—H12A	119.8
C3—C4—C5	102.95 (11)	C13—C12—H12A	119.8
O2—C4—H4A	112.3	C12—C13—C14	120.05 (15)
C3—C4—H4A	112.3	C12—C13—H13A	120.0
C5—C4—H4A	112.3	C14—C13—H13A	120.0
C6—C5—C4	101.12 (11)	C13—C14—C9	119.87 (14)
C6—C5—C1	114.18 (11)	C13—C14—H14A	120.1
C4—C5—C1	101.61 (11)	C9—C14—H14A	120.1
			
C3—O1—N1—C8	−147.02 (12)	N1—C1—C5—C4	67.40 (13)
C3—O1—N1—C1	−1.68 (13)	C2—C1—C5—C4	−37.18 (14)
C4—O2—N2—C6	0.13 (15)	O2—N2—C6—C7	−179.40 (12)
C8—N1—C1—C2	179.42 (13)	O2—N2—C6—C5	0.08 (16)
O1—N1—C1—C2	36.62 (13)	C4—C5—C6—N2	−0.24 (15)
C8—N1—C1—C5	72.72 (16)	C1—C5—C6—N2	−108.48 (14)
O1—N1—C1—C5	−70.08 (13)	C4—C5—C6—C7	179.23 (13)
N1—C1—C2—C3	−53.69 (12)	C1—C5—C6—C7	70.99 (17)
C5—C1—C2—C3	56.34 (13)	O1—N1—C8—O3	154.39 (12)
N1—O1—C3—C2	−34.33 (13)	C1—N1—C8—O3	15.5 (2)
N1—O1—C3—C4	74.07 (12)	O1—N1—C8—C9	−31.74 (18)
C1—C2—C3—O1	54.66 (12)	C1—N1—C8—C9	−170.68 (12)
C1—C2—C3—C4	−55.54 (13)	O3—C8—C9—C14	−30.7 (2)
N2—O2—C4—C3	110.46 (13)	N1—C8—C9—C14	155.66 (13)
N2—O2—C4—C5	−0.27 (14)	O3—C8—C9—C10	145.16 (15)
O1—C3—C4—O2	174.48 (10)	N1—C8—C9—C10	−28.4 (2)
C2—C3—C4—O2	−77.50 (14)	C14—C9—C10—C11	−0.5 (2)
O1—C3—C4—C5	−73.40 (13)	C8—C9—C10—C11	−176.30 (14)
C2—C3—C4—C5	34.62 (14)	C9—C10—C11—C12	−0.8 (2)
O2—C4—C5—C6	0.29 (13)	C10—C11—C12—C13	1.2 (2)
C3—C4—C5—C6	−116.25 (12)	C11—C12—C13—C14	−0.4 (2)
O2—C4—C5—C1	118.09 (12)	C12—C13—C14—C9	−0.9 (2)
C3—C4—C5—C1	1.56 (14)	C10—C9—C14—C13	1.3 (2)
N1—C1—C5—C6	175.33 (11)	C8—C9—C14—C13	177.27 (13)
C2—C1—C5—C6	70.76 (15)		

### Hydrogen-bond geometry (Å, º)

**Table:**

*D*—H···*A*	*D*—H	H···*A*	*D*···*A*	*D*—H···*A*
C4—H4*A*···O3^i^	1.00	2.50	3.2793 (18)	135
C5—H5*A*···O2^ii^	1.00	2.59	3.4017 (18)	138
C7—H7*B*···O2^ii^	0.98	2.56	3.3900 (19)	142
C7—H7*C*···O2^iii^	0.98	2.60	3.568 (2)	170
C11—H11*A*···O3^iv^	0.95	2.58	3.470 (2)	156

Symmetry codes: (i) −*x*+1/2, *y*−1/2, *z*; (ii) −*x*+1/2, *y*+1/2, *z*; (iii) *x*+1/2, −*y*+1/2, −*z*+1; (iv) −*x*+1, *y*−1/2, −*z*+1/2.
